# Articulatory Performance in Dysarthria: Using a Data-Driven Approach to Estimate Articulatory Demands and Deficits

**DOI:** 10.3390/brainsci12101409

**Published:** 2022-10-20

**Authors:** Mili Kuruvilla-Dugdale, Antje S. Mefferd

**Affiliations:** 1 Department of Speech, Language and Hearing Sciences, University of Missouri, Columbia, MO 65211, USA; 2 Department of Hearing and Speech Sciences, Vanderbilt University Medical Center, Nashville, TN 37232, USA

**Keywords:** articulatory kinematics, dysarthria, articulatory performance, assessment

## Abstract

This study pursued two goals: (1) to establish range of motion (ROM) demand tiers (i.e., low, moderate, high) specific to the jaw (J), lower lip (LL), posterior tongue (PT), and anterior tongue (AT) for multisyllabic words based on the articulatory performance of neurotypical talkers and (2) to identify demand- and disease-specific articulatory performance characteristics in talkers with amyotrophic lateral sclerosis (ALS) and Parkinson’s disease (PD). J, LL, PT, and AT movements of 12 talkers with ALS, 12 talkers with PD, and 12 controls were recorded using electromagnetic articulography. Vertical ROM, average speed, and movement duration were measured. Results showed that in talkers with PD, J and LL ROM were already significantly reduced at the lowest tier whereas PT and AT ROM were only significantly reduced at moderate and high tiers. In talkers with ALS, J ROM was significantly reduced at the moderate tier whereas LL, PT, and AT ROM were only significantly reduced at the highest tier. In both clinical groups, significantly reduced J and LL speeds could already be observed at the lowest tier whereas significantly reduced AT speeds could only be observed at the highest tier. PT speeds were already significantly reduced at the lowest tier in the ALS group but not until the moderate tier in the PD group. Finally, movement duration, but not ROM or speed performance, differentiated between ALS and PD even at the lowest tier. Results suggest that articulatory deficits vary with stimuli-specific motor demands across articulators and clinical groups.

## 1. Introduction

The examination of the articulatory subsystem is an important part of the clinical, perceptual-based speech assessment because its impairment may indicate pathological changes within the brain (e.g., neurological disease processes). Furthermore, it has been shown that articulatory impairments can significantly contribute to speech intelligibility loss in talkers with progressive dysarthria, e.g., [1,2,3]. Therefore, an assessment of the articulatory subsystem is critical to determine therapeutic intervention strategies and specify treatment targets.

For assessments of the articulatory subsystem in clinical settings, standardized reading passages (e.g., Grandfather passage; [4]) as well as sentence and/or word lists are commonly used. Some of these materials are phonetically balanced to test a wide range of articulatory movement patterns. However, the articulatory performance demands inherent to commonly used test stimuli are still poorly understood. This lack of knowledge prevents not only a systematic manipulation of articulatory performance demands during assessments but also hinders insights into articulatory mechanisms that underlie perceptible difficulties with the production of specific test stimuli. The former problem limits a clinician’s ability to quantify the severity of articulatory impairments accurately and reliably, which in turn negatively affects their ability to track articulatory performance changes over time. The latter problem hinders clinicians from deriving articulator-specific treatment targets based on the diagnostic findings.

### 1.1. Understanding and Leveraging Articulatory Demands of Stimuli in Dysarthria Assessments

The importance of understanding test stimuli demands was recognized a long time ago [5,6]. However, due to knowledge gaps about articulatory demands inherent to commonly used speech stimuli, maximum performance tasks, such as rapid syllable repetitions (i.e., DDK tasks) have enjoyed longstanding popularity (see [7] for an overview). The benefits of DDK tasks for clinical assessment are easy to discern because the articulatory demands are well understood and performance breakdowns are relatively straightforward to interpret (e.g., reduced repetition rate during /puh-puh-puh/ = slowness of lip + jaw complex; imprecise production of /kuh-kuh-kuh/ = undershoot of posterior tongue + jaw complex). Indeed, DDK rate alterations are deemed salient for the diagnosis of certain dysarthria types (e.g., slow DDK rates for spastic dysarthria). DDK-specific kinematic parameters are also known to achieve high discriminability within subgroups of talkers with amyotrophic lateral sclerosis (ALS) and Parkinson’s disease (PD) who are presumed to have distinct phenotypes [7,8]. Despite its usefulness, the articulatory demands of DDK tasks differ from those of running speech and assessment findings based on DDK tasks can be dissociated from those based on speech stimuli [9].

In the literature, few methods exist for quantifying articulatory demands in running speech. Using the developmental framework by Kent [10], such demands are determined based on articulatory motor adjustments tied to phoneme acquisition. With this approach, each phoneme gets a score through a tiered system that groups phonemes by the complexity of the articulatory movements required for their production and the age at which sounds are acquired. Individual phoneme scores are then totaled to determine the overall complexity of the utterance. The only kinematic study to have used the Kent [10] framework reported significantly smaller and slower jaw and tongue movements only for high complexity words in talkers with PD relative to controls [11]. Although Kent’s approach allows quantification of articulatory demands and shows promise to better detect and stage articulatory decline, some important shortcomings should also be noted. For example, the approach does not consider coarticulatory effects in connected speech. Coarticulation is known to impact articulatory movement patterns as it is an attempt to minimize articulatory effort [12,13]. Furthermore, it remains unclear if phonemes acquired earlier during speech development are motorically easier to execute than those acquired later, given the fact that mature talkers place phonemes in a rich phonetic environment while they exist in a constrained phonetic environment early on in speech development. Thus, the developmental perspective for estimating articulatory motor demands may not adequately capture the actual motor demands associated with mature speech performance.

In contrast to the segmental approach by Kent [10], Lehner and Ziegler [14] used non-linear gestural (NLG) scores derived from articulatory features extracted hierarchically from the gestural, syllabic, and lexical levels, to express articulatory complexity or ease [15]. Articulatory ease was computed based on how resistant words were to speech errors in talkers with acquired apraxia of speech. In talkers with dysarthria, Lehner and Ziegler [14] found a significant interaction between articulatory complexity and neighborhood frequency where motorically complex words with low frequency phonological neighbors were more intelligible than complex words with high frequency neighbors. Presumably, to aid the recognition of a word with many high frequency competitors, talkers need to produce fine phonetic distinctions to help distinguish it from its neighbors, which can be challenging for those with dysarthria to produce. So far, the NLG method is based on studies of German talkers and has not yet been tested in English-speaking individuals.

### 1.2. Range of Motion as an Important Articulatory Feature to Define Motor Performance Demand

The current study implemented a data-driven kinematic approach to determine articulatory demands. We chose to specifically focus on range of motion (ROM) as an articulatory feature that is of important clinical relevance. For example, ROM of the tongue + jaw complex has been shown to be directly associated with F1–F2 acoustic vowel space size, e.g., [16,17,18], which in turn has been shown to impact speech intelligibility, e.g., [19]. Although movement speed also has important diagnostic value for dysarthria, speed performance is challenging to interpret because speed often covaries with ROM [20]. A talker with a small ROM, for example, will likely also produce relatively low speeds; however, this talker may not be constrained in their ability to generate speed *per se*. In addition, speed changes are not necessarily associated with articulatory rate changes [21,22]. Therefore, ROM, but not speed, was selected as a feature to define the articulatory demands of target stimuli.

### 1.3. ROM Performance and Disease-Specific Deficits in Talkers with ALS and PD

Most talkers with dysarthria exhibit articulatory imprecision, which is often linked to insufficient articulatory ROM (i.e., undershoot, [23]). In the current paper, we chose to specifically focus on two clinical groups that have been studied extensively in isolation, however, rarely in comparison with each other: talkers with ALS and PD. The articulatory mechanisms underlying imprecise articulation are presumed to differ across the two clinical groups [23]. That is, in talkers with PD, articulatory imprecision is often conceptualized as the result of a general downscaling of articulatory movements (i.e., hypokinesia) in the presence of relatively adequate movement durations [23,24]. In contrast, slowness due to insufficient force generation is thought to result in articulatory undershoot and prolonged movement durations in talkers with ALS [25].

As to which articulators show the greatest ROM deficits, some studies suggest that all articulators are affected in PD, e.g., [24], while others implicate the tongue more than the lips and jaw, e.g., [26,27]. In talkers with ALS, however, the tongue is more affected than the lips and jaw, e.g., [28,29]; yet tongue ROM is not necessarily abnormally small in these talkers, e.g., [27]. Furthermore, jaw ROM can be abnormally large in talkers with ALS, e.g., [30], which has been commonly interpreted as a compensatory behavior in response to insufficient tongue ROM [2,28,31]. However, abnormally large jaw ROM has also been observed for utterances in which tongue ROM was unaffected [27].

### 1.4. Research Purpose and Hypotheses

This paper pursued two main research goals to address two major shortcomings of current assessment approaches of the articulatory subsystem: (1) to establish articulator-specific ROM demand tiers for a set of speech stimuli based on the articulatory performance of neurotypical controls and (2) to determine demand- and disease-specific articulatory performance characteristics in talkers with dysarthria due to ALS and PD across the established articulator-specific ROM demand tiers. To achieve the first aim, we used a data-driven approach to rank a variety of multisyllabic words into articulator-specific ROM demand tiers (i.e., low, moderate, high) based on the observed vertical ROM of the jaw (J), lower lip (LL), anterior tongue (AT), and posterior tongue (PT). For the second aim, we compared articulatory performance of talkers with ALS and PD with those of neurotypical controls across the established articulator-specific ROM demand tiers. In addition to the ROM measure, which was our primary focus, we also examined average speed and movement duration to capture potential performance tradeoffs (e.g., speed-accuracy tradeoffs).

The following hypotheses were tested:

(1) Both clinical groups were expected to increase ROM as a function of articulator-specific ROM demand; however, these within-group increases in ROM were expected to be smaller in magnitude than those of controls.
(2) Overall, both clinical groups were expected to exhibit deviant ROM performance when compared to controls with magnitudes of between-group differences varying across different ROM demands.
(3) Movement durations were expected to differentiate talkers with ALS and PD better than ROM and speed, regardless of the articulator.

## 2. Materials and Methods

### 2.1. Participants

This study was approved by the Institutional Review Boards of the University of Missouri (MU) and Vanderbilt University Medical Center (VUMC). Written consent was obtained, and all participants were compensated for their participation. Speech kinematic data were obtained as part of a larger project. The current study included data from 12 individuals with ALS (7 males, 5 females), 12 individuals with PD (6 males, 6 females), and 12 age- and sex-matched neurotypical controls (7 males, 5 females). The mean age of the ALS group was 65.99 years (SD = 9.85; age range = 47.4–80.2 years), of the PD group was 71.51 years (SD = 7.85; age range = 60.11–88.8 years), and of the control group was 64.14 years (SD = 10.76; age range = 51.4–82 years). All participants met the following inclusionary criteria: (a) monolingual speakers of American English, (b) no prior history of speech, language, or hearing impairments, (c) no neurosurgical treatment, including deep brain stimulation, (d) no cognitive impairment per self-report or a dementia diagnosis, (e) no hearing aids or prescription for hearing aids, and (f) no metal in the head and/or neck region, including pacemakers. The PD participants were all medicated 1–2 h prior to data collection. All the participants with ALS and PD as well as five of the control participants were tested at MU; the remaining seven controls were tested at VUMC.

Participants were screened using the Montreal Cognitive Assessment (MoCA [32] and the Mini-Mental State Examination (MMSE [33]) at MU and VUMC, respectively. Both screening tools assess cognitive functions, such as attention, memory, recall, language, and orientation, to provide an overall score out of 30. A score below 26 for the MoCA and below 24 for the MMSE indicates cognitive impairment [32,33].

Only the control participants tested at VUMC completed a pure tone hearing screening, and hearing sensitivity was assessed at the following frequencies: 500 Hz, 1000 Hz, 2000 Hz, and 4000 Hz. Results from this screening indicated that all the VUMC control participants could detect pure tones at or below 45 dB HL at the lower three frequencies. At 4 kHz, two participants had a hearing threshold above 45 dB HL bilaterally, while the rest of them had thresholds below 45 dB HL in both ears. While the ALS, PD, and control participants at MU did not complete a hearing screening, none of the participants showed signs of a hearing problem. In addition, all participants demonstrated the ability to follow instructions and conversations at typical loudness levels.

Sentence intelligibility and speech rate were calculated for all participants using all 11 sentences of the Speech Intelligibility Test (SIT [34]). Intelligibility scores were provided by the SIT software (Omaha, NE, USA) following orthographic transcriptions of read sentences by trained research assistants who were instructed to type the sentences exactly as they were heard. Percent intelligibility was calculated as the quotient of words correctly understood by the total number of words, multiplied by 100. Speaking rate was calculated as the quotient of the total number of words by the total duration in minutes (see Table 1 for participant information).

### 2.2. Experimental Stimuli

To classify stimuli based on articulatory motor demands using a data-driven approach, 20 words were chosen from the larger project. All selected words contained an initial bilabial or labiodental consonant, but the place of articulation of the final phoneme varied. Word length ranged from 2–4 syllables. Lexical and linguistic properties such as neighborhood density, phonotactic probability, and word frequency were also estimated for the selected words. Neighborhood density is defined as the number of words that are similar phonologically to the target word [35], and the average value for our stimulus set was 3.5 (SD = 5.34). Phonotactic probability is defined as the likelihood that a phonological segment will occur in a given position within a word [36], and the average probability of our stimulus set was 0.0006 (SD = 0.002). Word frequency reflects the distribution of words in spoken language [37], and the average value for our stimulus set was 2.08 (SD = 0.76). A detailed description of these properties and their calculations is available in [11].

### 2.3. Experimental Task

Participants read aloud each target word within the carrier phrase “Say ___ again” at a normal rate and loudness. A list of five target words was displayed at a time on a television monitor and readability was confirmed prior to data collection. Words in each list were consistent across participants, but their order was randomized. Between 5–10 repetitions of each list were recorded from all participants using a high-quality microphone placed approximately 20 cm away from the mouth. At MU, audio signals were recorded using a condenser microphone (Shure, Model PG42, Niles, IL, USA) and stored on a digital recorder (Marantz, Model PDM670, Eindhoven, The Netherlands). At VUMC, audio was collected using a lavalier condenser microphone (Audiotechnica, Model AT899, Stow, OH, USA) and a digital recorder (Tascam, Model DR-100KMII, Montebello, CA, USA).

### 2.4. Data Acquisition and Analysis

Data were recorded using two different 3D electromagnetic articulography devices, i.e., Wave (NDI, Waterloo, ON, Canada) and AG501 (Carstens Medizinelektronik, GmbH, Nelkenweg, Germany), at MU and VUMC, respectively. Precision and data from the two devices are comparable [11,38]. At both data collection sites, sensors were placed along the midsagittal plane on the anterior tongue (AT), posterior tongue (PT), lower lip (LL), and jaw center (J). Using non-toxic glue (Periacryl^®^90, Glustitch Inc., Delta, BC, Canada), sensors were placed 1 cm and 4 cm from the tongue tip for AT and PT, respectively, and on the vermillion border of the lower lip for LL. Small amounts of putty (Stomahesive, ConvaTec, Deeside, UK) held the sensor on the lower gumline below the central incisors for J. Three head reference sensors were placed on goggles for the AG501, and a single 5-degrees of freedom sensor was secured to the forehead via a headband for the Wave system. Kinematic data from both systems were head-corrected and smoothed using a 15 Hz low-pass filter. Further, data from the AG501 underwent an additional step, where the data were transposed into a head-based coordinate system, which is an automatic process for the Wave system. For the transposition, a recording was made while the participant held down on a bite plate housing three additional sensors [39]. This recording was then used for biteplate correction, to create a head-based coordinate system with the origin located just anterior to the jaw center sensor. This step also rendered the coordinates of the two articulography devices similar. Movement of the orofacial sensors were expressed in the x (lateral), y (vertical), and z (protrusive) dimensions relative to the reference head sensor(s). The sampling rate for the Wave system was 400 Hz, whereas for the AG501, the original sampling rate of 1250 Hz was down sampled to 250 Hz. The audio was synchronized with the kinematic data and was sampled at 22,000 Hz and 48,000 Hz for the Wave and AG501 systems, respectively.

SMASH (version 2.0, Boston, MA, USA) [40] was used to parse the target words from the carrier phrase using reliable kinematic landmarks. For all words, peak vertical displacement of the lower lip was used as the onset marker. For each final phoneme, peak displacement of the primary articulator in the y-dimension was used to mark word offset. Displacement peaks were marked in SMASH based on an algorithm that uses the zero-crossings of the velocity signal to identify displacement peaks and troughs. The vertical time histories of the parsed words were then used to extract kinematic metrics of interest, namely ROM, average speed, and movement duration for each articulator sensor.

Based on each sensor’s Euclidean distance, the relative distance from minimum to maximum in the dorsal-ventral dimension (i.e., y-dimension) was calculated to index ROM for each word. It should be noted that this approach provides a kinematic measure of ROM that should be conceptualized as an articulatory working space measure because it captures the movement extent from the positional minimum to the positional maximum within the target utterance. Although the ROM measure can be influenced by phoneme-specific vocal tract configurations as well as lexical stress demands, this measurement approach is well-suited for the purpose of this study because it quantifies word-specific motor demands. In addition, it is important to point out that the extracted ROM measures of the LL, AT, and PT included contributions of the jaw because the LL, AT, and PT signals were not decoupled from the jaw. A decoupling approach was not used in the current study because the ultimate goal of this work is to systematically assess ROM demand tiers in the clinic using auditory-perceptual ratings. Therefore, changes in vocal tract configurations (indexed by AT + J, PT + J, or LL + J ROM measures) were more appropriate than decoupled ROM measures of the LL, AT, and PT.

Average speed of the word-length utterance was derived by computing the first-order derivative of each sensor’s vertical time history based on its Euclidean distance from the head-based origin. Movement duration was calculated as the total time of sensor movement between the onset and offset of the target word.

## 3. Results

### 3.1. Articulatory-Specific ROM Demands Tiers Based on Performance of Controls

To establish data-driven ROM demand tiers, a linear mixed model analysis was conducted with all eight target words as the fixed effect and subject as the random effect by submitting the ROM of the J, LL, AT, and PT of controls to separate models (i.e., one model for each articulator). Pairwise comparisons were used to sort target words into articulator-specific ROM demand tiers. Target words with comparable ROM values were placed in the same ROM demand tier. Furthermore, target words with moderate ROM demands that did not elicit significantly larger ROM means than the smallest ROM demand tier as well as significantly smaller ROM means than the highest ROM demand tier were eliminated.

Table 2 lists the resulting target words, their assigned ROM demand tier, and the mean ROM (+/−SE) based on the control group’s ROM performance. Using only the ROM measures for the target words assigned to specific ROM demand tiers, a linear mixed model analysis was completed for each articulator to verify significant differences in ROM across tiers. These linear mixed models yielded a significant main effect of tier for all four articulators: J [*F*(5, 39.1) = 277.7, *p* < 0.001]; LL [*F*(3, 103.2) = 299.8, *p* < 0.001]; AT [*F*(4, 89.19) = 269.3, *p* < 0.001]; and PT [*F*(6, 47.59) = *p* < 0.001]. Pairwise comparisons confirmed that words in different tiers showed significant differences in ROM (*p* < 0.001). It should be noted that J ROM also significantly differed for “violinist” and “velocity” within tier 2 (*p* = 0.002) and for “metropolis” and “popsicle” within tier 3 (*p* < 0.001); however, their mean differences were much smaller than those across demand tiers. Finally, AT ROM of “velocity” was slightly but significantly smaller than AT ROM of “violinist” (Table 2, *p* = 0.048).

### 3.2. Articulatory Performance of Talkers with ALS and PD across Articulator-Specific ROM Demand Tiers

To determine between- and within group effects, a linear mixed model analysis was conducted with tier and group as fixed effects and subject as the random effect. Table 3 shows the findings of the linear mixed models (one model per articulator and kinematic measure), which revealed significant between-group and within-group findings as well as a significant group × tier interaction for all articulators (J, LL, AT, PT) and measures (ROM, average speed, duration). Post hoc analyses were conducted for between-group effects at each tier using a linear mixed model approach with group as the fixed effect and subjects as the random effect for each articulator and measure (Table 4). Pairwise comparisons were also completed for within-group effects using linear mixed models with group as the fixed effect and subject as the random effect (Table 5).

#### 3.2.1. ROM

**Figure 1:**
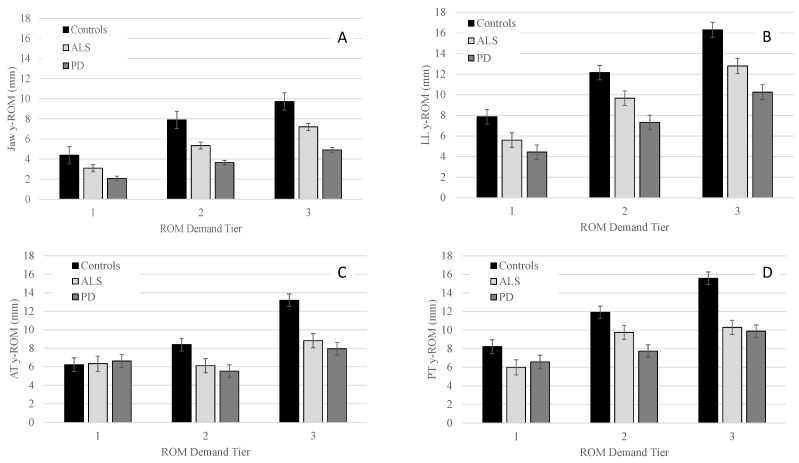
Group means (+/−SE) for y-ROM of the jaw (Panel (**A**)), lower lip (Panel (**B**)), anterior tongue (Panel (**C**)), and posterior tongue (Panel (**D**)) across all ROM demand tiers.

Panels A–D of Figure 1 show the ROM means (+/−SE) for each group as a function of the articulatory demand tiers. Significant main effects of group were found for each tier for J [Tier 1: *F*(2, 29.6) = 7.7, *p* = 0.002; Tier 2: *F*(2, 30.6) = 11.6, *p* < 0.001; Tier 3: *F*(2, 30.8) = 9.5, *p* < 0.001] and LL [Tier 1: *F*(2, 32.8) = 8.6, *p* < 0.001; Tier 2: *F*(2, 32.5) = 6.2, *p* = 0.005; Tier 3: *F*(2, 32.9) = 8.5; *p* = 0.001]. For the tongue, main effects of group were observed for tier 2 and tier 3, but not for tier 1 for AT [Tier 2: *F*(2, 29.7) = 4.7, *p* = 0.02; Tier 3: *F*(2, 29.9) = 12.8, *p* < 0.001] and PT [Tier 2: *F*(2, 31.8) = 6.3, *p* = 0.005; Tier 3: *F*(2, 31.7) = 15.5, *p* < 0.001].

Pairwise comparisons for J and LL revealed that the PD group had significantly smaller ROM than controls in all three tiers, with between-group differences becoming more pronounced as demands increased from tier 1 to tier 3. For both AT and PT, PD had significantly smaller ROM than controls in tier 2 and tier 3 with between-group differences being greater in tier 3 than tier 2 (Table 4).

By contrast, talkers with ALS only exhibited a significantly smaller ROM than controls in tier 3 for LL, AT, and PT. For J, the ALS group displayed significantly smaller ROM in tier 2 and tended to have smaller ROM in tier 3 relative to controls (*p* = 0.06). Talkers with PD and ALS did not differ significantly for any of the articulators in any of the three tiers (Table 4).

Within each group, the main effect of tier was significant for J ROM [Controls: *F*(2, 75.1) = 620.0, *p* < 0.001; PD: *F*(2, 135.3) = 471.2, *p* < 0.001; ALS: *F*(2, 130.0) = 523.0, *p* < 0.001]; LL ROM [Controls: *F*(2, 96.1) = 449.7, *p* < 0.001; PD: *F*(2, 106.8) = 328.4, *p* < 0.001; ALS: *F*(2, 106.8) = 328.4, *p* < 0.001]; AT ROM [Controls: *F*(2, 90.5) = 544.9, *p* < 0.001; ALS: *F*(2, 57.0) = 83.3, *p* < 0.001; PD: *F*(2, 124.9) = 183.0, *p* < 0.001], and PT ROM [Controls: *F*(2, 166.0) = 374.4, *p* < 0.001; ALS: *F*(2, 117.0) = 143.0, *p* < 0.001; PD: *F*(2, 195.7) = 95.0, *p* < 0.001]. Pairwise comparisons within each group revealed that all talkers significantly increased their ROM as a function of tier (see Table 5). For AT, the controls exhibited a similar increase in ROM as a function of articulatory demand. Furthermore, talkers with ALS produced significantly greater AT ROM in tier 3 relative to tiers 1 and 2; however, AT ROM did not significantly differ between tiers 1 and 2 in ALS (see Table 5). By contrast, talkers with PD produced significantly smaller AT ROM in tier 2 compared to tiers 1 and 3. However, they had significantly larger AT ROM in tier 3 than in tiers 1 and 2. Finally, Table 5 shows that controls increased ROM to a greater extent across tiers than talkers with PD and ALS for all articulators.

#### 3.2.2. Average Speed

**Figure 2:**
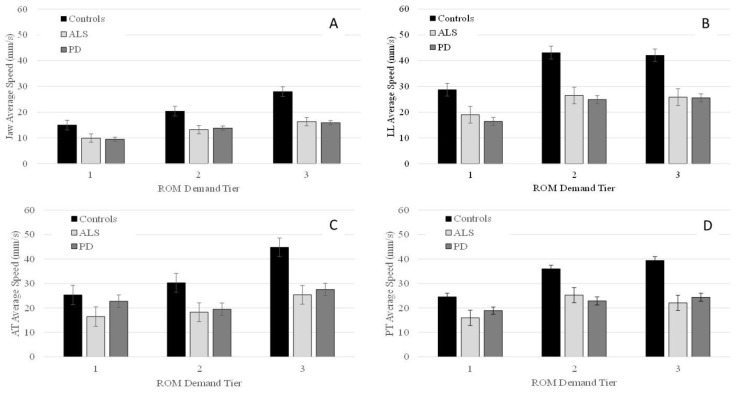
Group means (+/−SE) for average speed of the jaw (Panel (**A**)), lower lip (Panel (**B**)), anterior tongue (Panel (**C**)), and posterior tongue (Panel (**D**)) across all ROM demand tiers.

Panels A–D of Figure 2 show the group means for average speed in the y-dimension as a function of the demand tiers for all four articulators. Significant main effects of group were found for each tier for J [Tier 1: *F*(2, 30.9) = 5.4, *p* = 0.010; Tier 2: *F*(2, 30.7) = 6.5, *p* = 0.005; Tier 3: *F*(2, 30.8) = 10.8, *p* < 0.001]; LL [Tier 1: *F*(2, 32.3) = 7.1, *p* = 0.003; Tier 2: *F*(2, 32.7) = 12.1, *p* < 0.001; Tier 3: *F*(2, 32.8) = 10.4, *p* < 0.001]; and PT [Tier 1: *F*(2, 31.8) = 4.0, *p* < 0.029; Tier 2: *F*(2, 31.9) = 7.6, *p* < 0.002; Tier 3: *F*(2, 31.8) = 17.4, *p* < 0.001] For AT, significant main effects of group were observed for tier 2 and tier 3 [Tier 2: *F*(2, 30.0) = 3.4, *p* = 0.037; Tier 3: *F*(2, 29.9) = 10.8, *p* < 0.001], but not for tier 1.

Pairwise comparisons indicated that talkers with PD had significantly lower J and LL speed than controls in all three tiers with between-group differences in J speed becoming more pronounced as ROM demands increased (Table 4). For LL, between-group differences were greater for tiers 2 and 3 than tier 1. For AT, although main effects were significant for tiers 2 and 3, pairwise comparisons did not reveal any significant differences between talkers with PD and controls in tier 2. In tier 3, talkers with PD had significantly lower AT speeds than controls. For PT, pairwise comparisons showed that talkers with PD had significantly reduced speed than controls in tiers 2 and 3 (see Table 4). The between-group difference in PT speed became more pronounced as ROM demands increased from tier 2 to tier 3 (Table 4).

Talkers with ALS had significantly lower speed than controls in all three tiers for J, LL, and PT. Between group differences were more pronounced as ROM demands increased for J and PT. For LL, the between-group difference was greater for tiers 2 and 3 than tier 1. For AT, pairwise comparisons did not reveal any significant differences between ALS and controls in tiers 1 and 2, but in tier 3, talkers with ALS had significantly lower AT speed than controls (Table 4). Comparisons between talkers with ALS and talkers with PD were non-significant for all three tiers.

Within each group, the main effect of performance tier was significant for J [Controls: *F*(2, 82.3) = 525.2, *p* < 0.001; PD: *F*(2, 35.1) = 292.6, *p* < 0.001; ALS: *F*(2, 82.3) = 311.6, *p* < 0.001]; LL [Controls: *F*(2, 93.3) = 266.6, *p* < 0.001; PD: *F*(2, 136.0) = 245.2, *p* < 0.001; ALS: *F*(2, 119.0) = 69.1, *p* < 0.001]; AT [Controls: *F*(2, 50.0) = 394.2, *p* < 0.001; ALS: *F*(2, 13.6) = 91.5, *p* < 0.001; PD: *F*(2, 143.2) = 237.7, *p* < 0.001]; and PT [Controls: *F*(2, 63.5) = 451.0, *p* < 0.001; ALS: *F*(2, 100.3) = 140.6, *p* < 0.001; PD: *F*(2, 127.4) = 83.8, *p* < 0.001]. Pairwise comparisons within each group revealed that all talkers significantly increased J and PT speed as a function of ROM demand tier; however, the magnitude of change was greatest in the control group compared to the PD and ALS groups (Table 5). In all three groups, the only significant increase in LL speed was between tiers 1 and 2, but not between tiers 2 and 3. For AT, pairwise comparisons revealed that in controls AT speed increased significantly from tier 1 to tier 2 as well as from tier 2 to tier 3. Talkers with ALS produced significantly greater AT speed in tier 3 than in tier 2; however, their AT speed was not significantly different between tier 1 and tier 2. Finally, in talkers with PD, AT speed was significantly greater in tier 1 compared to tier 2 and in tier 3 compared to tier 2 (Table 5).

#### 3.2.3. Movement Duration

**Figure 3:**
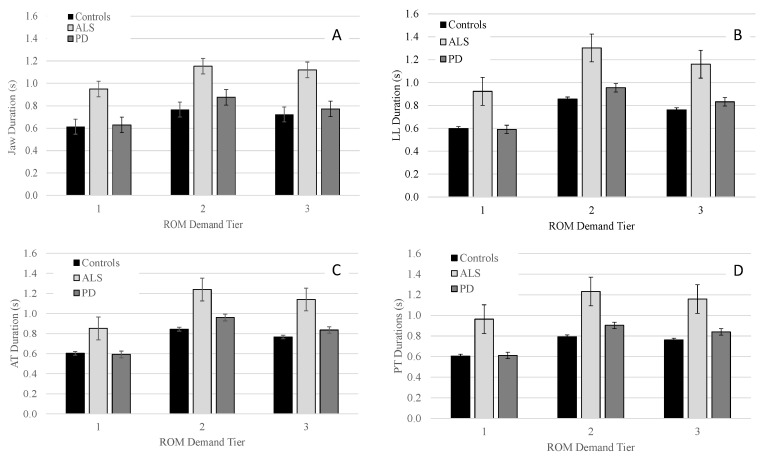
Group means (+/−SE) for movement duration of the jaw (Panel (**A**)), lower lip (Panel (**B**)), anterior tongue (Panel (**C**)), and posterior tongue (Panel (**D**)) across all ROM demand tiers.

Panels A–D of Figure 3 show the durations (+/−SE) for each group as a function of the articulatory demand tiers. Significant main effects of group were found for each tier for J [Tier 1: *F*(2, 29.9) = 9.5, *p* < 0.001; Tier 2: *F*(2, 30.3) = 7.8, *p* = 0.002; Tier 3: *F*(2, 30.1) = 9.1, *p* < 0.001]; LL [Tier 1: *F*(2, 31.8) = 8.6, *p* = 0.001; Tier 2: *F*(2, 32.5) = 7.6, *p* = 0.002; Tier 3: *F*(2, 32.7) = 8.1; *p* = 0.001]; AT [Tier 1: *F*(2, 30.2) = 7.1, *p* = 0.003; Tier 2: *F*(2, 28.4) = 6.3, *p* = 0.005; Tier 3: *F*(2, 29.4) = 7.7, *p* = 0.002]; and PT [Tier 1: *F*(2, 30.8) = 7.1, *p* = 0.003; Tier 2: *F*(2, 31.5) = 7.6, *p* = 0.002, Tier 3: *F*(2, 31.8) = 7.1, *p* = 0.003].

Pairwise comparisons indicated that for J, LL, AT, and PT talkers with ALS had significantly longer movement durations than talkers with PD and controls in all three tiers with between-group differences being more pronounced in tier 2 and 3 than tier 1 (see Table 4). One exception was the comparison for LL within tier 2 where talkers with ALS tended to have longer movement durations than talkers with PD; however, the statistical test did not reach significance (*p* = 0.062). Pairwise comparisons between talkers with PD and controls for movement duration of J, LL, AT, and PT did not reveal significant differences for any of the three tiers.

Within each group, the main effect of performance tier was significant for J [Controls: *F*(2, 98.1) = 304.1, *p* < 0.001; PD: *F*(2, 204.4) = 246.0, *p* < 0.001; ALS: *F*(2, 63.3) = 82.4, *p* < 0.001]; LL [Controls: *F*(2, 103.1) = 594.1, *p* < 0.001; PD: *F*(2, 136.6) = 583.0, *p* < 0.001; ALS: *F*(2, 71.6) = 414.7, *p* < 0.001]; AT [Controls: *F*(2, 98.0) = 445.8, *p* < 0.001; ALS: *F*(2, 58.3) = 387.5, *p* < 0.001; PD: *F*(2, 131.5) = 432.2, *p* < 0.001]; and PT [Controls: *F*(2, 73.0) = 458.8, *p* < 0.001; ALS: *F*(2, 59.7) = 238.2, *p* < 0.001; PD: *F*(2, 109.7) = 380.0, *p* < 0.001]. For J, LL, AT, and PT, pairwise comparisons within all three groups revealed that movement durations significantly increased from tier 1 to tier 2 and significantly decreased from tier 2 to tier 3; however, across all three groups, the increase in movement duration from tier 1 to tier 2 was greater in magnitude than the decrease in movement duration from tier 2 to tier 3 (see Table 5).

## 4. Discussion

The current paper pursued two main research goals to address two major shortcomings of current assessment approaches of the articulatory subsystem: (1) to establish articulator-specific ROM demand tiers for a set of speech stimuli based on the articulatory performance of neurotypical controls and (2) to determine demand- and disease-specific articulatory performance characteristics in talkers with dysarthria due to ALS and PD across the established articulator-specific ROM demand tiers. To achieve the first study goal, we used a data-driven approach to assign eight target words into three articulator-specific ROM demand tiers (i.e., low, medium, high). The articulators of interest were J, LL, AT, and PT. For the second study goal, we compared articulatory performance of talkers with PD, ALS, and controls across the established ROM demands. Although ROM performance was the primary focus of this study, we also examined corresponding speed performance, and movement durations to capture potential speed-accuracy tradeoffs.

The first hypothesis predicted all three groups to alter their ROM as a function of demand with the magnitude of ROM increase being greater in the control group than the ALS and PD groups. Our findings fully support this hypothesis for J, LL, and PT ROM performance, but only partially for AT ROM. Specifically, all three groups significantly increased their J, LL, and PT ROM as a function of demand tier with the magnitude of ROM increase being smaller for talkers with ALS and PD than controls. However, only the control group significantly increased AT ROM as a function of demand tier. Both clinical groups showed a significant increase in AT ROM from tier 2 to tier 3, but not from tier 1 to tier 2.

The second hypothesis predicted both clinical groups to exhibit deviant ROM performance compared to controls with the magnitude of these between-group differences expected to vary with ROM demands. Indeed, as predicted, talkers with ALS and PD differed from controls in their ROM in tier 3 more than in tier 1 and 2. However, significant between-group effects were in the same direction (i.e., reduced ROM of talkers with dysarthria) regardless of disease type, the articulator, and demand tier. Finally, the third hypothesis predicted that movement durations would differentiate talkers with ALS and PD better than ROM and speed performance, regardless of the articulator. This hypothesis was supported. In fact, movement duration was the only measure that yielded significant differences for comparisons between talkers with ALS and PD.

### 4.1. Hypothesis 1: Performance Changes across ROM Demand Tiers

Talkers with ALS and PD exhibited significantly reduced J, LL, and PT ROM performance in tier 1 when compared to control talkers; however, they were able to achieve the ROM required for tier 1 when producing the stimuli of tier 2. Similarly, despite their failure to produce adequately sized TT ROMs in tier 2, talkers with ALS and PD were able to accomplish such TT ROMs in tier 3. Such an observation is difficult to interpret; however, we speculate that talkers with dysarthria vary their articulatory strategies with articulatory demands of the utterance. For example, talkers may trade longer durations for larger ROM in utterances that require particularly large articulatory excursions. Similarly, in utterances with relatively low ROM demands, articulatory undershoot may reduce durational discrepancies in talkers with a limited ability to generate adequate force. Thus, our findings on performance changes across ROM demand tiers may indicate that talkers with dysarthria attempt to balance out their spatial and temporal performance constraints, likely in an effort to maximize speech intelligibility.

### 4.2. Hypothesis 2: Comparing ROM Performance across Groups

#### 4.2.1. Jaw Findings

In the current study, jaw ROMs of talkers with ALS were significantly reduced in the moderate demand tier compared to controls and tended to also be smaller in the low and high demand tiers. This finding was particularly surprising because Yunusova and colleagues [27] reported word-specific findings for talkers with ALS with significantly smaller jaw ROM for stimuli that required particularly large jaw ROM (i.e., “bad”, “cat”) and significantly larger jaw ROM for stimuli that required relatively small jaw ROM (i.e., “big”). Exaggerated jaw movements were also reported in other kinematic studies on talkers with ALS, e.g., [30,41]. The absence of abnormally large jaw ROM and a trend towards reduced jaw ROM in talkers with ALS in the current study suggest that jaw articulatory behavior can vary in these talkers. Future studies are warranted to determine the conditions under which specific jaw behaviors can be elicited and to understand the underlying mechanisms of such variable articulatory performance in talkers with ALS.

In talkers with PD, reports on jaw ROM have been mixed with several studies reporting reduced jaw ROM, e.g., [24,42] while others report no significant differences in jaw ROM, e.g., [11,41]. Findings on talkers with PD in the current study concur with previous reports of reduced jaw ROM. However, the mixed findings in the literature also suggest that stimuli-specific articulatory demands may elicit a wide range of jaw articulatory behaviors in talkers with PD. As mentioned above, future research is warranted to determine the factors that elicit the specific jaw articulatory behaviors in talkers with dysarthria.

#### 4.2.2. Lower Lip Findings

Lower lip findings parallel those of the jaw. However, it is important to note that the target words that make up each demand tier differ across the two articulators. Nevertheless, because the lower lip was not decoupled from the jaw, lower lip findings were influenced to some extent by jaw performance. Even with an impaired jaw, it is possible for talkers to achieve adequate lower lip ROM. For example, relative to controls, talkers with ALS showed significantly reduced jaw ROM for the moderate demand tier; however, there were no significant between-group differences in lower lip ROM for the moderate demand tier (Figure 1, Table 4). Thus, talkers with ALS likely increased the relative contribution of their lower lip to achieve adequate lower lip + jaw displacements at the moderate demand tier for the lower lip. Such motor equivalence has been reported in previous perturbation studies where the jaw was fixed with a bite block [43]. Because upper lip movements were not included in the kinematic analysis of the current study, it is unknown if talkers with ALS also adjusted their upper lip ROM disproportionally to compensate for the reduced jaw ROM. Interestingly, the high demand tier specific to the lower lip revealed significantly reduced lower lip ROM in talkers with ALS relative to controls whereas no significant differences between these two groups were observed for jaw ROM at the high demand tier. This observation is difficult to explain in context of the findings for the jaw and lower lip ROM at the moderate demand tiers and underscores the need for future studies with more fine-grained kinematic analyses of segment-specific performance within speech stimuli for the jaw as well as decoupled lower lip performance.

#### 4.2.3. Tongue Findings

Except for the moderate demand tier, findings for the anterior and posterior tongue ROM were similar for both clinical groups. That is, tongue ROM was significantly reduced relative to controls at the high demand tier but comparable at the low demand tier (see Figure 1, Table 4). Furthermore, no significant differences were observed between the two clinical groups. In one of the few available kinematic studies that compared tongue ROM between talkers with PD and ALS, tongue ROM was also reduced to similar extents in the two clinical groups relative to controls, at least for some of the speech stimuli [27]. Taken together, these findings suggest that talkers of both clinical groups can accommodate AT ROM demands when they are low. However, at high tongue ROM demands, talkers of both clinical groups exhibit significantly reduced AT ROM.

#### 4.2.4. Considering Findings across All Articulators

Considering the ROM performance of all articulators, talkers with PD showed more difficulty than talkers with ALS to comply with increasing ROM demands. This observation is congruent with the notion that hypokinesia is a cardinal symptom of PD but not ALS. However, it is difficult to determine any differential impairment of the tongue and lower lip ROM based on the findings of the current study because the ROM of these articulators can also be greatly impacted by the jaw ROM. For example, if the jaw is not adequately lowered in talkers with PD, the tongue ROM may also be restricted in these talkers. Thus, even a decoupling approach to observe tongue and lower lip movements independent of the jaw may not necessarily provide a full understanding of the articulator-specific constraints in each clinical group because of biomechanical influences of one impaired articulator on the others.

### 4.3. Hypothesis 3: Disease-Specific Performance Profiles

Of the three examined kinematic measures, movement duration was the only measure that showed significant differences between the two clinical groups even at the lower demand tiers. ROM and speed measures did not differentiate ALS and PD in any of the three tiers. Given the fact that talkers with ALS and PD do exhibit distinct dysarthria symptoms when considering their auditory-perceptual speech features, other articulatory performance characteristics may yield significant differences between these two talker groups. For example, a recent acoustic study by [44] examined consistency and coordination to identify articulatory phenotypes distinct to ALS and PD based on syllable repetition tasks (i.e., puh-tuh-kuh). They found that in the early disease stages, ALS had significantly more impaired coordination than PD, and PD showed more impairment in speed than ALS. In their study, speed was calculated based on the mean slope of the second formant, which may not be linearly related to actual articulatory speed and may explain discrepancies between our findings for speed and theirs. It may also explain why another acoustic study showed events in the spatial domain to be more sensitive than durational measures in differentiating mild ALS and PD [45].

When examining within group performance, ALS and PD had different kinematic profiles as tier demands increased. That is, the difference in ROM and speed between talkers with PD and controls increased as ROM demands increased. However, movement durations remained comparable to that of the controls across all ROM demands. A similar profile has been reported previously for J and LL movements among talkers with PD by [24]. The proportional reduction of ROM and speeds commonly observed in talkers with PD are thought to occur due to a general downscaling of articulatory performance, e.g., [5]. This contrasts with the kinematic profile of talker with ALS where speed and hence durational performance deficits are disproportionately greater than ROM performance deficits. Especially in the lower tiers, talkers with ALS were able to achieve ROM demands while underperforming in speed and duration. Thus, despite non-significant findings for between group comparisons of ROM and speed, in context of each other, these two kinematic measures can differentiate talkers with ALS and PD: dysarthria in PD is characterized by hypokinesia whereas dysarthria is ALS is characterized by speed constraints.

### 4.4. Clinical Implications for the Assessment of Articulatory Performance

Existing perceptual studies suggest that tongue height regulation is affected early in ALS and that it continues to be the main articulatory deficit as intelligibility worsens, although the evidence for it is mixed [2,46]. In talkers with PD, there is a predominance of manner errors from incomplete closure and continual air emission, which are thought to occur due to articulatory undershooting [47]. Our findings for tongue articulatory performance across ROM demand tiers suggest that in both clinical groups tongue articulatory impairments may only be detectable when test stimuli elicit high tongue ROM demands while lower lip and jaw impairments may already be observable at relatively low ROM demands. Furthermore, perceptual speech features associated with temporal performance deficits (e.g., prolonged phonemes) may help distinguish dysarthria in ALS and PD even when ROM demands are low. Spatial deficits (e.g., articulatory imprecision) may prevail over temporal deficits in talkers with PD, whereas temporal deficits may dominate in talkers with ALS.

### 4.5. Limitations and Future Directions

For the current study, we decided to consider the entire word to characterize articulatory performance demands. The ROM measure is therefore influenced by lexical stress demands as well as phoneme-specific demands. This is important to keep in mind because findings of deviant articulatory performance in the current study cannot be linked to specific phoneme productions. Instead, our approach offers insight intp altered articulatory working space in the vertical dimension across the articulators (jaw, lower lip, anterior and posterior tongue). In the future, segmental kinematic data could also be examined to gain more insights in segment-specific articulatory deficit among clinical groups. For example, specific segments within each stimulus may be particularly challenging to articulate, which could be marked (i.e., underlined) so that the examiner can specifically focus on these segments and better interpret perceived articulatory breakdowns during the assessment.

For more clarity about articulator-specific impairment profiles, tongue and lip movements should be tested independent of the jaw. This could be done by asking talkers to hold a bite block between their teeth while producing specific utterances. However, the downside of such an approach is that it becomes more difficult to understand the articulator-specific mechanisms that underlie reduced speech intelligibility in unconstrained running speech tasks. Specifically, talkers with dysarthria may achieve adequate ROM of the tongue + jaw complex but not the tongue independent of the jaw. Thus, despite reduced independent tongue ROM, they may produce perceptually adequate utterances. To gain insights in disease-related articulatory impairments as well as their functional significance, kinematic measures may need to be extracted from a variety of speech tasks and include decoupled tongue and lower lip performance as well as kinematics of compound movements (i.e., tongue + jaw, lower lip + jaw). Ultimately, auditory-perceptual evaluations of articulatory performance, which were not included in the current study, need to be combined with the kinematic findings to determine the clinical value of assessing speech performance across ROM demand tiers in talkers with dysarthria.

## 5. Conclusions

The current study is a starting point for a more systematic assessment of articulatory performance in talkers with dysarthria based on knowledge about the articulatory demands of test stimuli. The findings of the current study suggest that in talkers with ALS and PD jaw and lower lip impairments (i.e., reduced ROM) can already be identified in stimuli with low ROM demands, whereas impairments involving the tongue require higher ROM demands to emerge. However, it remains difficult to tease out articulatory performance capacities because talkers have the option to trade spatial for temporal demands and vice versa. Talkers with PD underperform on ROM demands even when ROM demands are low but exhibit adequate temporal performance while talkers with ALS achieve ROM demands at lower demand tiers but demonstrate compromised temporal performance. Such disease-specific patterns are likely due to the distinct differences in the underlying pathophysiology.

## Figures and Tables

**Table 1:** Demographic and clinical details of participants in the ALS and PD groups.

S. ID	Age/Sex	MoCA Scores (Out of 30)	Speech Intelligibility (%)	Speaking Rate (Words per Minute)
ALS1	67.5/M	19	99.00	164.90
ALS2	56.1/M	28	100.00	231.25
ALS3	80.2/M	26	99.39	145.17
ALS4	71.4/M	26	99.09	149.36
ALS5	47.4/M	26	53.61	55.10
ALS6	75.0/M	19	56.13	103.73
ALS7	66.4/M	23	98.79	188.34
ALS8	58.11/F	29	90.91	115.62
ALS9	69.10/F	27	93.67	106.69
ALS10	74.11/F	22	49.13	58.70
ALS11	72.1/F	22	92.56	128.23
ALS12	54.4/F	26	99.85	152.68
PD1	71.9/M	28	93.09	180.14
PD2	88.8/M	27	94.72	165.91
PD3	71.1/M	24	99.09	173.16
PD4	77.0/M	25	98.54	186.51
PD5	69.7/M	25	99.82	178.67
PD6	78.1/M	20	97.44	145.62
PD7	70.5/F	27	99.64	166.14
PD8	75.5/F	19	93.27	191.95
PD9	65.8/F	26	99.45	167.76
PD10	60.8/F	24	97.27	213.36
PD11	68.8/F	27	99.82	184.11
PD12	60.11/F	25	99.55	188.52

ALS = amyotrophic lateral sclerosis, PD = Parkinson’s disease, M = Male, F = Female, MoCA = Montreal Cognitive Assessment.

**Table 2:** Control group’s ROM means for speech stimuli in their respective range of motion (ROM) demand tiers.

Variable	Tier	Word	Mean	SE
Jaw y-ROM (mm)	1	Festivities	4.26	0.86
		Princess	4.34	0.87
	2	Violinist	7.18	0.87
		Velocity	7.97	0.85
	3	Metropolis	9.18	0.89
		Popsicle	10.26	0.89
LL y-ROM (mm)	1	Princess	8.02	0.61
	2	Festivities	11.69	0.67
		Preposterous	12.67	0.64
	3	Velocity	16.25	0.67
AT y-ROM (mm)	1	Princess	6.44	0.86
	2	Festivities	8.33	0.80
	3	Velocity	12.69	0.82
		Metropolis	13.38	0.83
		Violinist	13.56	0.83
PT y-ROM (mm)	1	Festivities	8.40	0.67
		Princess	9.60	0.70
	2	Metropolis	11.60	0.65
		Preposterous	12.80	0.65
	3	Velocity	15.80	0.64

**Table 3:** Statistical findings of linear mixed models.

Variable	Effect	Jaw	Lower Lip	Anterior Tongue	Posterior Tongue
		*F*_df_; *p* Value			
y-ROM	Tier	F(2, 475.5) = 1455.6, <0.001	F(2, 308.8) = 966.3, <0.001	F(2, 324.7) = 594.4, <0.001	F(2, 386.8) = 595.8, <0.001
	Group	F(2, 29.3) = 15.6, <0.001	F(2, 32.2) = 11.8, <0.001	F(2, 31.8) = 4.1, 0.027	F(2, 31.7) = 9.5, <0.001
	Tier × Group	F(4, 466.2) = 59.4, <0.001	F(4, 306.1) = 12.8, <0.001	F(4, 320.6) = 57.6, <0.001	F(4, 387.1) = 52.32, <0.001
Average Speed	Tier	F(2, 576.9) = 788.0, <0.001	F(2, 362.8) = 416.7, <0.001	F(2, 39.9) = 516.6, <0.001	F(2, 377.4) = 587.2, <0.001
	Group	F(2, 30.5) = 9.9, <0.001	F(2, 32.8) = 12.3, <0.001	F(2, 31.4) = 5.0, 0.013	F(2, 32.0) = 10.5, <0.001
	Tier × Group	F(4, 568.5) = 49.0, <0.001	F(4, 361.2) = 18.3, <0.001	F(4, 41.9) = 48.9, <0.001	F(2, 372.2) = 54.8, <0.001
Duration	Tier	F(2, 571.6) = 442.3, <0.001	F(2, 332.4) = 1185.8, <0.001	F(2, 367.4) = 995.3, <0.001	F(2, 303.2) = 670.4, <0.001
	Group	F(2, 30.5) = 8.7, <0.001	F(2, 32.4) = 7.9, <0.001	F(2, 30.2) = 8.0, =0.002	F(2, 31.4) = 7.3, =0.003
	Tier × Group	F(4, 565.2) = 12.2, <0.001	F(4, 330.5) = 13.03, <0.001	F(4, 363.1) = 20.1, <0.001	F(4, 301.5) = 10.8, <0.001

**Table 4:** Results of post hoc analyses for tier x group interactions (i.e., between-group effects).

Measure	Articulator	Tier 1						Tier 2						Tier 3					
		Controls-PD		Controls-ALS		ALS-PD		Controls-PD		Controls-ALS		ALS-PD		Controls-PD		Controls-ALS		ALS-PD	
		*M* Diff (SE)	*p*	*M* Diff (SE)	*p*	*M* Diff (SE)	*p*	*M* Diff (SE)	*p*	*M* Diff (SE)	*p*	*M* Diff (SE)	*p*	*M* Diff (SE)	*p*	*M* Diff (SE)	*p*	*M* Diff (SE)	*p*
y-ROM (mm)	J	1.8 (0.5)	0.001	n.s.		n.s.		3.8 (0.8)	<0.001	2.1 (0.8)	0.037	n.s.		4.9 (1.1)	<0.001	n.s.		n.s.	
	LL	3.2 (0.8)	<0.001	n.s.		n.s.		4.0 (1.2)	0.004	n.s.		n.s.		6.4 (1.6)	<0.001	4.4 (1.6)	0.029	n.s.	
	AT	n.s.		n.s.		n.s.		2.9 (1.0)	0.017	n.s.		n.s.		5.3 (1.1)	<0.001	4.4 (1.2)	0.003	n.s.	
	PT	n.s.		n.s.		n.s.		4.1 (1.2)	0.004	n.s.		n.s.		6.4 (1.3)	<0.001	5.8 (1.3)	<0.001	n.s.	
Average Speed (mm/s)	J	5.2 (1.8)	0.018	4.7 (1.8)	0.037	n.s.		6.3 (2.1)	0.014	6.5 (2.1)	0.011	n.s.		12.5 (3.2)	0.001	12.7 (3.2)	0.001	n.s.	
	LL	11.6 (3.2)	0.003	9 (3.3)	0.027	n.s.		17.1 (3.9)	<0.001	16.1 (3.9)	<0.001	n.s.		16.8 (4.2)	0.001	16.4 (4.2)	0.001	n.s.	
	AT	n.s.		n.s.		n.s.		n.s.		n.s.		n.s.		17.7 (4.5)	0.001	19.7 (4.9)	0.001	n.s.	
	PT	n.s.		7.3 (2.7)	0.034	n.s.		13.1 (3.7)	0.004	12.2 (3.8)	0.009	n.s.		16.4 (3.4)	<0.001	18.8 (3.5)	<0.001	n.s.	
Duration (s)	J	n.s.		−0.3 (0.1)	0.002	−0.3 (0.1)	0.003	n.s.		−0.4 (0.1)	0.002	−0.3 (0.1)	0.031	n.s.		−0.4 (0.1)	0.001	−0.3 (0.1)	0.007
	LL	n.s.		−0.3 (0.1)	0.004	−0.3 (0.1)	0.003	n.s.		−0.4 (0.1)	0.003	−0.4 (0.1)	0.014	n.s.		−0.4 (0.1)	0.002	−0.3 (0.1)	0.011
	AT	n.s.		−0.2 (0.1)	0.007	−0.2 (0.1)	0.007	n.s.		−0.4 (0.1)	0.005	n.s.		n.s.		−0.4 (0.1)	0.002	−0.3 (0.1)	0.013
	PT	n.s.		−0.3 (0.1)	0.008	−0.3 (0.1)	0.008	n.s.		−0.4 (0.1)	0.003	−0.4 (0.1)	0.012	n.s.		−0.4 (0.1)	0.004	−0.3 (0.1)	0.02

ROM = range of motion, *M* Diff = mean difference, SE = standard error, J = jaw, LL = lower lip, AT = anterior tongue, PT = posterior tongue, n.s. = non-significant.

**Table 5:** Results of post hoc analyses for tier x group interactions (i.e., within-group effects).

Group		Controls				ALS				PD			
Within-Group		Tier 1 vs. Tier 2		Tier 2 vs. Tier 3		Tier 1 vs. Tier 2		Tier 2 vs. Tier 3		Tier 1 vs. Tier 2		Tier 2 vs. Tier 3	
		*M* Diff (SE)	*p*	*M* Diff (SE)	*p*	*M* Diff (SE)	*p*	*M* Diff (SE)	*p*	*M* Diff (SE)	*p*	*M* Diff (SE)	*p*
y-ROM (mm)	J	−3.5 (0.14)	<0.001	−1.8 (0.13)	<0.001	−2.3 (0.13)	<0.001	−1.8 (0.11)	<0.001	−1.6 (0.07)	<0.001	−1.3 (0.1)	<0.001
	LL	−4.3 (0.22)	<0.001	−3.9 (0.35)	<0.001	−3.7 (0.25)	<0.001	−3.5 (0.22)	<0.001	−2.9 (0.17)	<0.001	−2.8 (0.21)	<0.001
	AT	−1.9 (0.34)	<0.001	−4.9 (0.16)	<0.001	n.s.		−2.7 (0.23)	<0.001	1.0 (0.2)	<0.001	−2.3 (0.12)	<0.001
	PT	−3.7 (0.23)	<0.001	−4 (0.25)	<0.001	−3.7 (0.26)	<0.001	−0.8 (0.28)	0.028	−1.2 (0.15)	<0.001	−1.9 (0.25)	<0.001
Average Speed (mm/s)	J	−5.4 (0.34)	<0.001	−7.6 (0.42)	<0.001	−3.3 (0.31)	<0.001	−3.1 (0.24)	<0.001	−4.4 (0.25)	<0.001	−2.1 (0.3)	<0.001
	LL	−14.4 (0.7)	<0.001	n.s.		−7.5 (0.6)	<0.001	n.s.		−8.5 (0.5)	<0.001	n.s.	
	AT	−5.0 (0.9)	<0.001	−14.5 (0.7)	<0.001	n.s.		−7.1 (0.6)	<0.001	3.3 (0.7)	<0.001	−8.1 (0.4)	<0.001
	PT	−11.8 (0.47)	<0.001	−3.0 (0.63)	<0.001	−9.0 (0.57)	<0.001	3.2 (0.69)	<0.001	−3.9 (0.41)	<0.001	−1.5 (0.61)	0.04
Duration (s)	J	−0.2 (0.01)	<0.001	0.1 (0.01)	0.004	1.1 (0.01)	<0.001	0.1 (0.01)	0.002	−0.2 (0.01)	<0.001	0.1 (0.01)	<0.001
	LL	−0.3 (0.1)	<0.001	0.1 (0.1)	<0.001	−0.4 (0.1)	<0.001	0.1 (0.1)	<0.001	−0.4 (0.1)	<0.001	0.1 (0.1)	<0.001
	AT	−0.2 (0.1)	<0.001	0.1 (0.1)	<0.001	−0.4 (0.1)	<0.001	0.1 (0.1)	<0.001	−0.4 (0.1)	<0.001	0.1 (0.1)	<0.001
	PT	−0.2 (0.1)	<0.001	0.1 (0.1)	<0.001	−0.3 (0.1)	<0.001	0.7 (0.1)	<0.001	−0.3 (0.1)	<0.001	0.1 (0.1)	<0.001

ROM = range of motion, *M* Diff = mean difference, SE = standard error, J = jaw, LL = lower lip, AT = anterior tongue, PT = posterior tongue, n.s. = non-significant.

## Data Availability

The data presented in this study are available from the corresponding author [M.K.-D.] upon reasonable request.
